# “Transition from children’s to adult services for adolescents/young adults with life-limiting conditions: developing realist programme theory through an international comparison”

**DOI:** 10.1186/s12904-020-00620-2

**Published:** 2020-07-30

**Authors:** Helen Kerr, Kimberley Widger, Geraldine Cullen-Dean, Jayne Price, Peter O’Halloran

**Affiliations:** 1 School of Nursing and Midwifery, Queen’s University Belfast, Medical Biology Centre, Lisburn Road, Belfast, BT9 7BL Northern Ireland; 2 University of Toronto, Lawrence S Bloomberg John Hopkins University Baetjer Memorial Library, The Hopsital for Sick Children, Toronto, Canada; 3 The Hospital for Sick Children, Toronto, Canada; 4 Faculty of Health, Social Care and Education, Kingston and St George’s University, London, UK

## Abstract

**Background:**

Managing transition of adolescents/young adults with life-limiting conditions from children’s to adult services has become a global health and social care issue. Suboptimal transitions from children’s to adult services can lead to measurable adverse outcomes. Interventions are emerging but there is little theory to guide service developments aimed at improving transition. The **T**ransition to **A**dult **S**ervices for **Y**oung Adults with **L**ife-limiting conditions (TAYSL study) included development of the TASYL Transition Theory, which describes eight interventions which can help prepare services and adolescents/young adults with life-limiting conditions for a successful transition. We aimed to assess the usefulness of the TASYL Transition Theory in a Canadian context to identify interventions, mechanisms and contextual factors associated with a successful transition from children’s to adult services for adolescents/young adults; and to discover new theoretical elements that might modify the TASYL Theory.

**Methods:**

A cross-sectional survey focused on organisational approaches to transition was distributed to three organisations providing services to adolescents with life-limiting conditions in Toronto, Canada. This data was mapped to the TASYL Transition Theory to identify corresponding and new theoretical elements.

**Results:**

Invitations were sent to 411 potentially eligible health care professionals with 56 responses from across the three participating sites. The results validated three of the eight interventions: early start to the transition process; developing adolescent/young adult autonomy; and the role of parents/carers; with partial support for the remaining five. One new intervention was identified: effective communication between healthcare professionals and the adolescent/young adult and their parents/carers. There was also support for contextual factors including those related to staff knowledge and attitudes, and a lack of time to provide transition services centred on the adolescent/young adult. Some mechanisms were supported, including the adolescent/young adult gaining confidence in relationships with service providers and in decision-making.

**Conclusions:**

The Transition Theory travelled well between Ireland and Toronto, indicating its potential to guide both service development and research in different contexts. Future research could include studies with adult service providers; qualitative work to further explicate mechanisms and contextual factors; and use the theory prospectively to develop and test new or modified interventions to improve transition.

## Background

Transition from children’s to adult services has emerged in the last decade as a global health and social care issue. The ‘transition issue’ relates to young adults experiencing difficulties engaging with adult services after graduating from children’s services, typically when the young adult is 18 years old [1]. A lack of engagement with adult services can result in measurable adverse outcomes such as non-adherence to treatment and loss to follow up, in addition to adverse social and educational outcomes [2]. The lack of engagement may be the result of inadequacies in transition planning or a lack of transition preparation while the adolescent is in pediatric care [3]. The rationale for the transition issue is multifaceted with causes related to a disjointed transition process [4], adolescents/young adults experiencing difficulties adjusting to differences in the culture between children’s and adult services [5], the ending of long-standing relationships with children’s service providers, and loss of services [6]. Although a poor transition to adult care may be associated with poor clinical outcomes, higher costs to both the health system and the family as well as low levels of patient and family satisfaction [7], an international cross-jurisdictional policy scoping review focusing on identifying health system strategies that support transition to adult care in nine countries, found that the United Kingdom and Australia were the only countries which demonstrated efforts to develop transition to adult service strategies [7].

Transition from children’s to adult services is defined as the “purposeful, planned process that addresses the medical, psychosocial and educational/vocational needs of adolescents/young adults with chronic physical and medical conditions as they move from child-centred to adult-oriented health care systems” [8, p. 570]. This transition process may start at different ages and a variety of terms may be used to refer to this population. As graduation from childrens to adult services is recommended to occur when the adolescent legally becomes an adult at 18 years, for the purposes of this paper, we distinguish ‘adolescents’ as individuals between 10 and 17 years, and ‘young adults’ as referring to individuals aged 18–25 years [9]. The broad terminology ‘adolescent/young adult’ refers to individuals aged between 10 and 25 years. It is suggested the transition process should commence from the second decade of life, if not before [10] with 25 years identified as the maximum age to transfer to adult care [11].

The transition process has evolved in recent decades in response to the increasing numbers of young people graduating into adulthood as a result of improved health services, advances in medical treatment strategies and earlier detection of medical conditions [12]. Improved survival rates are also seen in the cohort of adolescents/young adults with life-limiting and life-threatening conditions [13]. There are four broad categories of life-limiting conditions affecting children and young people [14] (Table 1), with the ‘Directory of Life-Limiting Conditions in Children’ listing almost 400 diseases [15]. International studies vary in their estimated prevalence rates for pediatric life-threatening conditions. A Canadian study found a prevalence of 2.3 + .011 per 10,000 [16], whilst in England, United Kingdom, estimates are 32 per 10,000 of the population related to the number of children and young people up to 19 years living with a life-limiting condition in 2009/10 [13].

**Table 1:** Categories of life-limiting in children [14]

**Category 1:** Conditions for which curative treatment has failed e.g. cancer.	
**Category 2:** Conditions associated with periods of normal childhood activities, which usually require long periods of intensive treatment, but which are often associated with premature death e.g. cystic fibrosis, muscular dystrophy.	
**Category 3:** Progressive conditions without curative option where treatment may ameliorate the condition, which may extend over a number of years e.g. Battens disease.	
**Category 4:** Conditions with severe neurological disability that may deteriorate unpredictably but are not usually considered progressive e.g. multiple disabilities such as following severe brain or spinal injury, severe cerebral palsy.	

Transition interventions should aim to bridge the potential gap between child and adult services [17]. Transition planning should involve evidence-based interventions which lead to improved patient centred outcomes such as the adolescent/young adult increasingly taking responsibility for engaging with services providers, adhering to treatment strategies and contributing to their disease management plan [18]. Outcomes can be improved with the introduction of good transition programmes [2]. In recent years, benchmarks for a best practice approach for a range of medical conditions include recognition and support for a gradual process to transition, moving the management of a health condition to the adolescent/young adult, coordination of children’s and adult teams, inclusion of parents/carers in the process, assessing the adolescent/young adults transition readiness and involvement of the General Practitioner (family physician) [17]. The National Institute for Health and Care Excellence (NICE) [19] published five recommendations for a successful transition which includes using a person-centred approach and children’s and adult service working in an integrated way; transition planning; support before transfer; support after transfer and finally, a supporting infrastructure. Together for Short Lives, a national charity in the United Kingdom, published a Framework for Transition to Adult Services which outlines a three phased approach to care specifically in teenagers and young adults with life-limiting conditions; preparing for adulthood, preparing to move on, and settling into adult services [20].

### Development of the TASYL transition theory for adolescents/young adults with life-limiting conditions

**Fig. 1:**
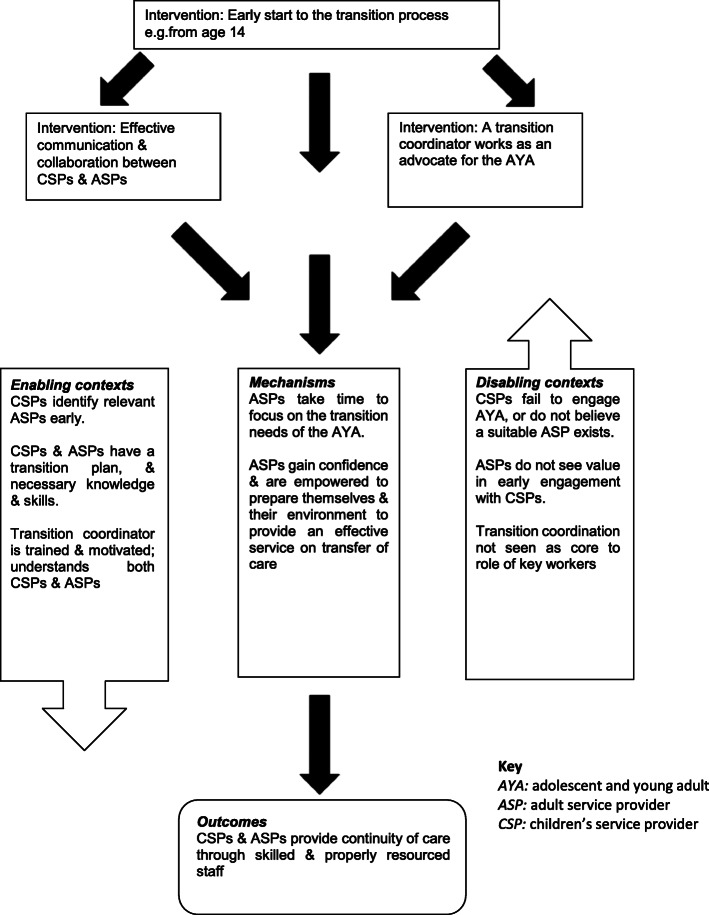
Preparing services for a successful transition: an integrated transition model

**Fig. 2:**
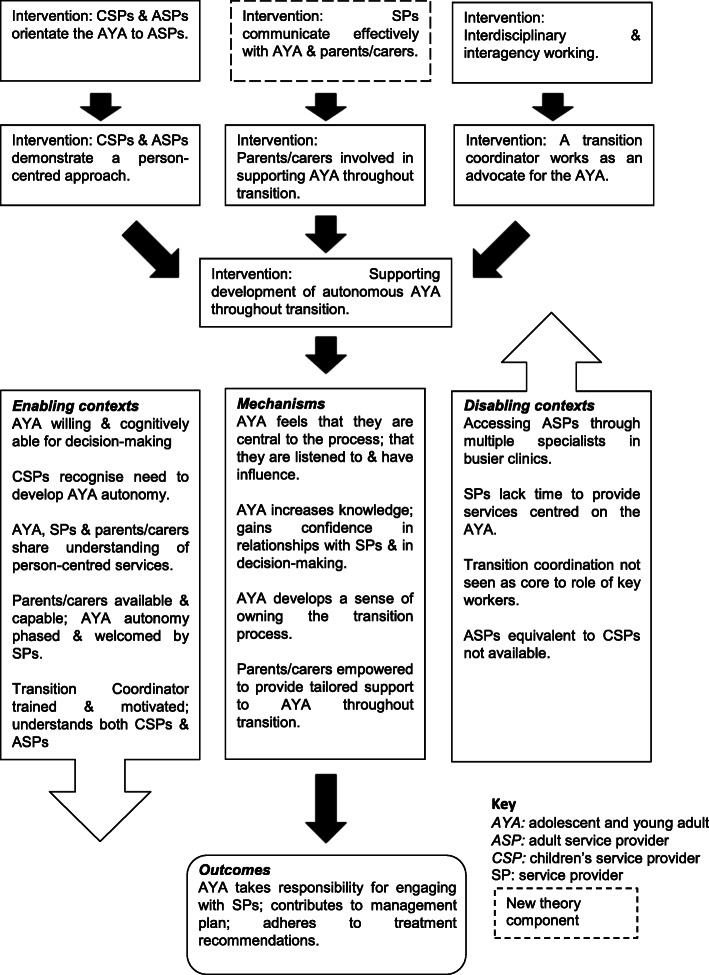
Preparing an adolescent/young adult for a successful transition: an integrated transition model

Transition theory was developed from a realist literature review [21] followed by observational research in the island of Ireland (the geographical term describing both Northern Ireland and the Republic of Ireland) (**T**ransition to **A**dult **S**ervices for **Y**oung Adults with **L**ife-limiting conditions (TAYSL study) using a realist evaluation design [18]. Realist evaluation seeks to determine ‘what works, for who, under what circumstances, and how’ [22], p. 1. The theory is expressed in the form of context-mechanism-outcome configurations, which provide a comprehensive explanation of how interventions function. The context may include the individuals, the relationships, the setting and infrastructure [23]. As each context is different, it is crucial to identify the contextual variations which help or hinder a successful transition. Mechanisms are the means by which interventions lead to outcomes, and are typically related to the reasoning, perceptions or beliefs of those involved in the programme [24]. The TASYL study identified eight key interventions considered to contribute to a successful transition to adult services for adolescents/young adults with life-limiting conditions (Table 2). The integration of the components of interventions, context, mechanism and outcome are presented in two transition models (Figs. 1 and 2), one focused on how services and organisations can prepare for the adolescent/young adult transitioning and the second focused on preparing the adolescent/young adult for transition. The models presented in this paper have been updated with the results of the present study with changes highlighted in Figs. 1 and 2. These models identify and integrate the crucial ingredients of a successful transition to adult services for adolescents/young adults with life-limiting conditions [18].

**Table 2:** Eight interventions related to a successful transition to adult services [18]

1. Early start to the transition process	
2. Effective communication and collaboration to joint working between children’s and adult services	
3. Orientation of the young adult to adult services	
4. The engagement of a transition coordinator	
5. Interdisciplinary and interagency joint working	
6. Developing the young adults’ autonomy throughout the transition process	
7. Service providers demonstrating a person-centred approach to care	
8. Involvement of parents/carers

We aimed to assess the usefulness of the TASYL Transition Theory in identifying interventions, mechanisms and contextual factors associated with a successful transition from children’s to adult services for adolescents/young adults through a critical comparison with the results of a research study undertaken in Toronto, Canada. This built on an existing partnership between the School of Nursing and Midwifery, Queen’s University Belfast and Lawrence S. Bloomberg Faculty of Nursing, University of Toronto, which share research interests and expertise in palliative care for children and young adults.

## Methods

### Survey

Phase one of the TASYL study in Ireland was a survey of children’s and adult services in health, social, educational and charitable organisations, using a questionnaire to gather descriptive data on the organisational approach to the transition to adult services for adolescents/young adults with life-limiting conditions in the island of Ireland. The survey results are reported elsewhere [1]. The 20 item questionnaire included open, closed and Likert-type questions. Questions focused on the type of transition services provided, the approach to managing transition, the organisational factors that contributed to the effectiveness of the transition process and the profile of service users. While the TASYL study included data collection from both childrens and adult services in health, social, educational and charitable organisations, the study in Toronto focused on collecting data only through pediatric health care centres. The survey was adapted in 2015 for use in Toronto based on a review of the literature with a particular focus on research conducted in Canada and the expertise of the study team and collaborators at each of the three sites. Items in the survey were also made more specific to services provided at the three data collection sites in Toronto (see Supplemental File 1 for survey items). The purpose of the survey in Toronto was to gather descriptive data on transition services from the perspective of health and social care professionals who provide care to adolescents with life-threatening conditions.

### Setting and sample

The survey was distributed through three institutions that provide care to adolescents with life-threatening conditions in Toronto, Canada: Hospital for Sick Children (SickKids), Holland Bloorview Kids Rehabilitation Hospital, and Emily’s House Children’s Hospice. Ethical approval for the study was secured at each site prior to proceeding with the study. At SickKids, a Good2Go (transition) Program was introduced in 2006 which provides consultation and support for transition to the complex care population, many of whom have life-threatening conditions [25]. At Holland Bloorview Kids Rehabilitation Hospital, a LIFEspan (transition) service is available to support adolescents/young adults with childhood-onset disability, which also includes non life-threatening conditions [26]. Emily’s House Children’s Hospice opened in 2013 and is a residential hospice for children with life-threatening conditions [27]. Emily’s House is part of the Philip Aziz Centre for Hospice Care, which provides non-medical care to both children and adults and is integrated into the patient’s professional health care team [28]. Services are provided through Philip Aziz within the patient’s home, while Emily’s House provides care to children 0–19 years of age within the residential hospice.

Health and social care professionals at the three institutions were eligible to take part in the study if they provided care to adolescents with life-threatening conditions who may need to be transitioned to adult services in the future or who have cared for adolescents with life-threatening conditions who are in the process or have already transitioned to adult services. At each site, co-investigators identified potentially eligible health and social care professionals, however, participants self-selected whether or not they provided care to children with conditions that fell into any of the four broad categories detailed by Together for Short Lives [14](Table 1). An email of invitation to complete the survey was forwarded by a manager. The email included a weblink to the survey which included a consent form. All surveys were anonymous. Two reminder/thank you emails were sent at one week and two weeks after the initial invitation. Once the survey was completed, participants were given information about the option of registering to be entered into a draw for one of five gift cards for $20(CAN) each.

### Data analysis

Responses to the survey were summarised descriptively with frequencies and proportions or means and standard deviations depending of the level of data. KW carried out the initial collation and analysis of the survey data. HK and PO’H then independently considered the survey data using a mixed methods sequential explanatory design, starting with the quantitative (numeric) data, then moving to the qualitative (text) data to help explain, or elaborate on, the quantitative results [29]. In the first phase we assessed the frequencies with which characteristics of the transition process were identified; then we read and re-read the qualitative data from the open-ended questions to identify common themes and consider how these illuminated the results of the quantitative analysis. We then discussed the results of this intitial analysis in relation to the Transition Theory, aiming to assess how far the data supported, refuted, modified or added to the the eight interventions and the overall models. This analysis was then critically evaluated by the other authors and final models agreed.

## Results

The results section presents the response rate to the survey and demographic information on the participants. Data from the survey were mapped to the eight interventions identified in the TASYL transition theory highlighting where the data supports and refutes the theory and if new theoretical elements emerged. For ease of reference, relevant sections and questions in the survey are highlighted when reporting results.

Data were collected from July 2015 to May 2016. Invitations were sent to 411 potentially eligible health and social care professionals in three organisations. There was a 100% organisational response rate. There were 56 individual responses from across the three participating sites indicating a 13.6% individual response rate. Most of the participants (71%) were employed at SickKids, 21% at Holland Bloorview and 5% at Emily’s House. Missing data accounts for the remaining percentage. Just over half (54%) of the total participants worked in both inpatient and outpatient settings. Participants included 22 physicians (39%), 19 nurses (34%), 6 social workers (11%), and 9 others (16%), which included occupational therapists, physical therapists, and therapeutic recreation specialists. In terms of years of experience working with adolescents with life-threatening conditions, 10 (18%) had less than 6 years, 17 (30%) had 6 to 10 years, and 28 (50%) had more than 10 years. One third of participants indicated they had transitioned more than 20 adolescents with life-threatening conditions to adult services over the last 5 years, while 40% indicated that they expected to transition more than 20 adolescents in the next 5 years.

### Intervention 1: early start to the transition process

Participants were asked to identify the three most important factors in promoting a successful transition from a list of five options provided with the option of adding a factor to the list (Section C, question 1). 82% stated an *‘early introduction of the transition process to the adolescent/family.’* Recommended improvements in the transition process included a consistent approach to early planning for transition and engaging adult services earlier in the process.

“Consistent early planning for transition.” (Participant #22, Nurse)

Participants reported a mean age of 15.9 years and a range of 12 to 20 years for the start of the transition process and a mean age of 19.2 years and range of 17 to 26 years for completion of the transfer to adult services.

When asked about the rationale for the absence of organised transition services, which would include an early start to the process, a lack of funding and time were outlined.

“Lack of funding and time to develop [transition services].” (Participant #21, Physician)

### Intervention 2: effective communication and collaboration to joint working between children’s and adult services

Participants were asked to identify the most commonly used model of transition to adult services from five approaches (Section B, question 5). This question invited participants to rank on a 5 point Likert scale, how seldom (rated 1) to frequently (rated 5) they used five models of transition. The most frequently used approach reported related to the development of good channels of communication and sharing of information between children’s and adult services, with 47% selecting 4 or 5. A range of strategies to promote effective communication between these services were reported to be used such as case conferences, joint clinics between services, and comprehensive referral letters.

“…we connect with the accepting [adult] team to provide extensive verbal and written handover.” (Participant #35, Physician)

“Adult team has a specific transition clinic and teens are seen in that clinic several times before integrating into the regular adult clinic.” (Participant #31, Nurse)

However, despite effective communication between children’s and adult services being the most commonly used model of transition, it was also reported to be a challenge.

“We are not always welcomed by [the] adult clinic.” (Participant #27, Social Worker)

“Adult services are not always open to meetings.” (Participant #30, Nurse)

Another barrier in developing good channels of communication between children’s and adult services related to a lack of time identifying that a joint appointment would be useful in preparing the adolescent/young adult for the transfer to adult services if time was available.

“Joint appointment with family medicine. Challenge though because no time” (Participant #21, Physician)

As well, 29 participants (52%) indicated that one of the most challenging barriers to a successful transition was the lack of an identified health care provider in the adult system who was available to provide care.

“There is no equivalent [expert] in the adult services world..” (Participant #4, Physician)

### Intervention 3: orientation of the adolescent/young adult to adult services

Several participants indicated that they used specific strategies such as a transition orientation day jointly organised by the adult and children’s team, to orient the adolescents to adult services (Section B, question 1, qualitiative responses). Further strategies included transition discussions between the service provider and young adult and a tour of the adult centre in advance of the transfer.

“I meet with youth to discuss differences between adult and pediatric services…” (Participant #10, Nurse)

“…ensure that a tour is provided to the adult centre at [the] last visit.” (Participant #26, Nurse)

A challenge reported by 25% of participants related to children’s service providers limited knowledge of adult services, which resulted in not being able to comprehensively orientate adolescents/young adults to the new system (Section C, question 2). There were no reports of adult service providers offering to address orientation deficits for children’s service providers.

“I don't believe I have a good enough understanding of the adult health care system to adequately [counsel] families with skill-building to transition in to this system.” (Participant #23, Nurse)

### Intervention 4: the engagement of a transition coordinator

Many participants (55%) indicated that the involvement of a key coordinator in the transition process was a key factor in promoting a successful transition (Section C, question 1). Participants were asked to rank how often a key coordinator was used in the transition process using a 5 point Likert scale with 1 indicating seldom used and 5 indicated frequently used. Only 7 (13%) indicated that they frequently used a key coordinator in the transition process and many (48%) identified a lack of coordinated care as a key barrier to a successful transition. There were concerns expressed regarding which health and social care professional could take responsibility for the transition coordinator role with some participants indicating that a number of professionals were required to support the adolescent/young adult in negotiating the transition process, rather than a single person.

“Not sure who would take this role on. Doctors and nurses [have] no time.” (Participant #21, Physician)

“Due to complexity, usually involves multiple people to manage transition.” (Participant #23, Nurse)

### Intervention 5: interdisciplinary and interagency joint working

Threaded throughout many responses was the importance of an interdisciplinary team approach to transition in children’s services within and between departments. The narrative included health and social care professionals working closely with members of different teams with examples provided such as team meetings and summary documentation from all health disciplines when the young adult transfers to adult services.

A number of participants emphasised a team approach to the transition process with 45% of participants indicating that the inclusion of all services and agencies was a key factor in a successful transition (Section C, question 1).

“We have a team of therapists that help with the [transition] process, not one individual is responsible for the whole piece, we all play a role.” (Participant #54, Occupational Therapist)

### Intervention 6: developing the adolescent/young adults’ autonomy throughout the transition process

Some participants stated adolescents/young adults were supported to become more responsible for their care. Of those who considered this strategy relevant to the cohort of adolescent/young adults they provided services for, 32% indicated a 4 or 5, on the 5 point Likert scale with 1 being seldom used and 5 being frequently used (Section B, question 5). Strategies to promote autonomy and increase confidence included encouraging the adolescent/young adult to book clinic appointments, supporting them to make decisions regarding their care, questions increasingly being directed towards the adolescent rather than the parent/carer, and the offer of lone consultations to adolescents.

“Encouraged to give graded responsibility to the teenager rather than disempowering or doing everything for them. Allowing [the] teenager to speak and take the lead.” (Participant #11, Physician)

“Mostly we focus on preparing the teen[ager] to take over their healthcare.” (Participant #5 Physician)

“Enhancing their confidence by offering them choices and putting them in a leadership role during every intervention.” (Participant #48, Other Health Professional)

As outlined by one participant, advocacy skills and autonomy were promoted through the use of specific tools:

“MyHealth Passport, 3 Sentence Summary, Alien Test ('If aliens kidnapped your parents tomorrow, how would you get your medications?')” (Participant #2, Physician).

One participant indicated that role-play might be an additional tool to support preparation for transition.

“In my view, our youth are still underprepared for what meets them in the adult world. There ought to be a program designed just for role-play in terms of how to prepare oneself for what lies ahead.” (Participant #48, Other Health Professional)

The role of parents/carers in this process was also recognised by a number of participants in gradually transferring the autonomy from the parent/carer to the adolescent/young adult.

“We strive to help the clients in our program develop strong self-advocacy skills. Parents are part of the process in the sense of helping to facilitate the transfer of responsibility of health care decision making from the parent to the client.” (Participant #55, Physical Therapist)

“Parents are helped/encouraged to relinquish some of their control.” (Participant #5 Physician)

Although the role of promoting the adolescents/young adults’ autonomy involves parents/carers relinquishing some control, one health and social care professional indicated that it can be a challenge for parents/carers.

‘Parents resist it (child becoming autonomous).’ (Participant #47, Nurse)

### Intervention 7: service providers demonstrating a person-centred approach to care

As the three organisations in this study were providing services to adolescents under 18 years, it was evident from responses the focus was more on family centred care, rather than person-centred care. There were examples provided on how family centred care is delivered such as family team meetings, family clinical meetings and family health teams and also a focus on educating the family on resources required in the transition process.

“Consistent family clinic meetings to facilitate the [transition] process” (Participant #54 Occupational Therapist)

“Try to educate the family and provide the resources they will need in transition.” (Participant #49, Physical Therapist)

One participant felt adult service providers were not investing in family centred care.

“Limited investment from adult providers to child and family centred care.” (Participant #27 Social Worker)

### Intervention 8: involvement of parents/carers

Parents were reported to be very involved in the transition process while the adolescent was in children’s services.

“Parents are always involved in the process through their presence at clinic visits and interactions with paediatric and healthcare professionals. In this way we encourage their awareness of and engagement with the need for transition, especially during the early [stages].” (Participant #5, Physician)

Participants were asked to indicate on a 5 point Likert scale how seldom (rated 1) to frequently (rated 5) they helped the parent/caregiver to acquire skills and support to use adult services effectively (Section B, question 5). Only 5 (9%) of participants frequently used this as an intervention to support the transition process.

One participant highlighted that parents have expressed serious concerns regarding the transition process with some fearing grave outcomes such as their child dying as a result of a poor transition, highlighting the uncertainty of the continuation of services which meet the adolescents/young adults needs, when transferred to adult services.

“Parents worry that [their] child will die because they are transferred.” (Participant #15, Physician)

## Discussion

Overall, the study findings lend support for the TASYL Transition Theory originally developed based on a literature review and surveys conducted in Ireland. Specifically, the Transition Model on ‘Preparing services for a successful transition’ (Fig. 1) was supported by the study findings in relation to two of the three interventions: early start to the transition process, and effective communication and collaboration between children’s and adult service providers. However, most participants did not use a transition coordinator, an intervention included in both models, and some saw this role as better shared among a number of professionals. This was an interesting finding as a transition coordinator and the role of a key worker to specifically support the young adult with a life-limiting condition to transition to adult services is recommended [30, 31]. Components of the enabling and disabling contexts were supported by the study findings, however, context related to the transition coordinator was not relevant when a transition coordinator is not used or not available to be used. Further research may be needed to identify additional interventions or enabling contexts that need to be in place if responsibility for transition coordination is shared across multiple people rather than a specific key worker.

The Transition Model on ‘Preparing an adolescent/young adult for a successful transition’ (Fig. 2) was supported by study findings in relation to all of the interventions except *demonstrating a person-centred approach.* This intervention was not specifically described but may well be approximated by the focus on family centred care. Given that all of the participants worked in pediatric settings where a family centered approach would be expected, this finding is not surprising. If a similar survey was completed by adult service providers, who typically focus on person-centered care, it is possible that the findings would be different. Perhaps a personalised approach inclusive of family, though defaulting to the young person themselves, is appropriate which involves the young adult being recognised as an individual [32]. One new intervention was evident in the data, namely *effective communication between healthcare professionals and the adolescent/young adult and their parents/carers.* It could be argued that this is implicit in the interventions already identified but nevertheless it does seem to be an important intervention in its own right.

In terms of contexts, children’s service providers recognising the need to develop the adolescent/young adults’ autonomy and service providers lacking time to provide services centred on the adolescent/young adult were both supported. Some mechanisms were also supported, including and the adolescent/young adult gaining confidence in relationships with service providers and in decision-making.

### Strengths and limitations

Our Canadian data came from only three institutions in Toronto, all of them children’s services, which limits the generalisability of the findings. In particular, we were not able to validate the mechanisms leading to outcomes in adult services as no adult service providers were included. Rather, we had to rely on inferences about adult services from data provided by children’s service providers. The survey method also reduced our ability to thoroughly investigate possible mechanisms at work – these being more easily identified through qualitative interviews. Nevertheless, we received 56 responses providing rich data from a broad cross-section of professionals with considerable clinical experience. Conducting the survey in another country and health system allowed us to test whether the Transition Theory would remain relevant in a different context. The realist approach to data collection and analysis is a strength of this study. The foundational work that led to this study provided an evidence-based starting point for theory development. Realist evaluation, although challenging in its complexity, is a theory-driven and theory creating methodology, and so well-suited to research seeking to test and develop theory [22, 23].

## Conclusions

Transition models on how organisations can prepare for the adolescent/young adult transitioning and on preparing the adolescent/young adult for transition was broadly supported by our data, with one new intervention (effective communication between healthcare professionals and the adolescent/young adult and their parents/carers) identified. The theory, developed in Ireland, seemed applicable in Toronto, indicating its potential to guide both service development and research in different contexts. Future research should include adult service providers along with children’s services in order to more effectively validate theory in relation to adult services. It could include qualitative work to further explicate the mechanisms and contextual factors that lead to the model outcomes, and what aspects of interventions actively trigger the desired mechanisms. The theory could also be used prospectively to help develop and test new or modified interventions to improve transition, perhaps combining testing of effectiveness in a randomised controlled trial with qualitative approaches in a process evaluation within a realist evaluation approach to provide greater explanatory power.

## Supplementary information

**Additional file 1.** Transition to Adult Services by Youth with Life-Threatening Conditions: a Survey for Health Professionals.

## Data Availability

The datasets during and/or analysed during the current study available from the corresponding author on reasonable request.

## Abbreviations

TASYL study: The Transition to Adult Services for Young Adults with Life-limiting conditions study
