# Supplementary material for: “Transition from children’s to adult services for adolescents/young adults with life-limiting conditions: developing realist programme theory through an international comparison”
Source: BMC Palliat Care. 2020 Jul 30;19:115. doi: 10.1186/s12904-020-00620-2 (PMC7393825; doi:10.1186/s12904-020-00620-2)
Supplement: Supplementary file 1 — Additional file 1. Transition to Adult Services by Youth with Life-Threatening Conditions: a Survey for Health Professionals. [file 12904_2020_620_MOESM1_ESM.docx]

**Transition to Adult Services by Youth with Life-Threatening Conditions:**

**A Survey for Health Professionals**

We are interested in the current transition process of youth aged 16 or older, with life-threatening conditions as defined below:

Pediatric life-threatening conditions (LTC) include those for which curative treatments may be feasible but may fail, or those for which a cure is not possible and from which an affected child is expected to die. They are frequently chronic complex conditions with significant impact upon the lives of the child and family (PedPalASCNET 2014).

For all items in the survey please respond in terms of the group of patients who would be considered to have a LTC according to this definition.

# SECTION A: ABOUT YOUR SERVICE USERS

**1. Which of the following categories do the youth with a LTC using your service fall into?**

We are using the widely accepted categories for illness trajectory in LTC recommended by the charity ‘Together for Short Lives.’ ([www.togetherforshortlives.org.uk](http://www.togetherforshortlives.org.uk))

**Check all that apply**

□ Category 1 – Conditions for which curative treatment may be feasible but can fail.

*Examples: Irreversible organ failures of heart, liver, kidney; cancer.*

□ Category 2 – Conditions where premature death is inevitable.

*Examples: Cystic fibrosis, Duchenne muscular dystrophy*.

□ Category 3 – Progressive neurological conditions without curative treatment options.

*Examples: Batten disease, mucopolysaccharidoses.*

□ Category 4 - Irreversible but non-progressive conditions causing severe disability, leading to susceptibility to health complications and likelihood of premature death.

*Examples: severe cerebral palsy, multiple disabilities, such as following brain or spinal cord injury, complex health care needs, high risk of an unpredictable life-threatening event or episode*.

**□** Other – (Please describe) ____________________________________________________

**2. Approximately how many youth with LTC do you provide care to on a monthly basis?**

□ 1 - 5

□ 6 - 10

□ 11 - 20

□ more than 20

**3. Approximately how many youth with LTC have you followed that have transitioned to adult services in the last 5 years?**

□ 1 - 5

□ 6 - 10

□ 11 - 20

□ more than 20

**4. Looking at your current caseload, approximately how many youth with LTC do you expect will need to transition to adult services in the next 5 years?**

□ 1 - 5

□ 6 - 10

□ 11 - 20

□ more than 20

**5. What percentage of these patients would you estimate to be cognitively impaired?**

□ <10%

□ 10-49%

□ 50-90%

□ >90%

**6. At what age do youth followed by your service usually:**

|  | **Age in Years** |
| --- | --- |
| 1. Begin the process of transition |  |
| 1. Formally transfer to adult services |  |
| 1. Complete the process of transition |  |

**7. Do you ever continue to provide care to youth within children’s services beyond the age of 18 /19 years?**

**□** Yes

□ No

**If yes, why does this happen?**

**__________________________________________________________________________________________________________________________________________________________________________**

# SECTION B: YOUR APPROACH TO MANAGING TRANSITION FOR YOUTH WITH LTC

**1. What resources do you use when preparing youth with LTC to transition to adult care?**

**□** SickKids Good 2 Go Program

□ Holland Bloorview LifeSpan Program

□ Service or program specific transition initiatives

□ Other: please specify

**2. Is there a transition policy and/or strategy in your service?**

**□** Yes If you have a policy that you are willing to share – please email it to kim.widger@sickkids.ca

□ No

**3. Is your service currently developing strategies, policies or processes related to the transition process?**

**□** Yes

□ No

**4. Has there been a formal or informal evaluation of the transition processes in your service?**

**□** Yes - If yes, what was evaluated and what did you find? _______________________

□ No

**5. In items ‘a’ to ‘g’ below, we describe various approaches to managing transition. Please indicate how frequently you use each approach (where 1 = ‘seldom used’ and 5 = ‘frequently used’) and provide brief examples.**

1. **Develop good channels of communication and share information between child and adult services (e.g., case conferences, joint clinics, detailed transfer summary)**

| **Seldom Used** | **1** | **2** | **3** | **4** | **5** | **Frequently Used** |
| --- | --- | --- | --- | --- | --- | --- |

**Examples: __________________________________________________________________________________________________________________________________________________________________________**

1. **Develop transition services as an extension of children’s services, or jointly between child and adult providers (for example transition or adolescent clinics)**

| **Seldom Used** | **1** | **2** | **3** | **4** | **5** | **Frequently Used** |
| --- | --- | --- | --- | --- | --- | --- |

**Examples:**

**__________________________________________________________________________________________________________________________________________________________________________**

1. **Help the youth acquire the skills and support systems necessary to use adult care effectively**

Indicate not applicable if the majority of your patients do not have the cognitive capacity to develop skills to use adult care.

**Not applicable** **0**

| **Seldom Used** | **1** | **2** | **3** | **4** | **5** | **Frequently Used** |
| --- | --- | --- | --- | --- | --- | --- |

**Examples:**

**__________________________________________________________________________________________________________________________________________________________________________**

1. **Help the parent/caregiver acquire the skills and support systems necessary to use adult care effectively**

| **Seldom Used** | **1** | **2** | **3** | **4** | **5** | **Frequently Used** |
| --- | --- | --- | --- | --- | --- | --- |

**Examples:**

**__________________________________________________________________________________________________________________________________________________________________________**

1. **Identify a key professional/worker who can manage the transition process for or with the youth/parent/caregiver**

| **Seldom Used** | **1** | **2** | **3** | **4** | **5** | **Frequently Used** |
| --- | --- | --- | --- | --- | --- | --- |

**Examples:**

**__________________________________________________________________________________________________________________________________________________________________________**

1. **Other approach**

| **Seldom Used** | **1** | **2** | **3** | **4** | **5** | **Frequently Used** |
| --- | --- | --- | --- | --- | --- | --- |

**Please briefly describe any other approach taken by your service:**

**__________________________________________________________________________________________________________________________________________________________________________**

1. **If there are no organised transition service available please provided any comment on why no services are available.**

**__________________________________________________________________________________________________________________________________________________________________________**

**6. How often is parent input actively sought as part of the transition process in your service?**

| **Seldom** | **1** | **2** | **3** | **4** | **5** | **Frequently** |
| --- | --- | --- | --- | --- | --- | --- |

**If parents are involved, please describe how:**

**__________________________________________________________________________________________________________________________________________________________________________**

**7. Advance care planning is a process of discussions between families and health care providers about preferences for care, treatments and goals in the context of the patient’s current and anticipated future health. It may include preparation of an advance directive or other documents that reflect health care decisions. How often do you have advance care planning discussions with the youth/parents as part of the transition process?**

| **Seldom** | **1** | **2** | **3** | **4** | **5** | **Frequently** |
| --- | --- | --- | --- | --- | --- | --- |

**If you do not tend to have these discussions with youth/parents, please indicate the reasons (check all that apply).**

**□** It is not my role to have these discussions

□ I do not have the knowledge/comfort level with discussing this topic

**□** I refer youth/families to PACT for these discussions

□ I feel these discussion should be held by the adult providers

**□** I don’t think youth/families want to have these discussions

□ Other: please describe

**__________________________________________________________________________________________________________________________________________________________________________**

**8. How often do you involve the Paediatric Advanced Care Team (PACT) or other palliative care team as part of the transition process?**

| **Seldom** | **1** | **2** | **3** | **4** | **5** | **Frequently** |
| --- | --- | --- | --- | --- | --- | --- |

**Why or why not?**

**__________________________________________________________________________________________________________________________________________________________________________**

**9. Where do youth with LTC transitioning out of your service go to for adult services? (check all that apply)**

□ Adult sub-specialty physician/team

□ Family Practitioner

□ CCAC – home care

□ Adult Hospice

□ Other – (Please describe) __________________________________________________

# SECTION C: ORGANISATIONAL FACTORS AFFECTING TRANSITION

**1. In your experience, what are the most important factors to promote successful transition to adult services for youth with LTC? Please choose up to 3 that you see as most important.**

□ Early introduction of the transition process to the youth/family

□ Effective communication between health professionals and the youth/family

□ Inclusion of all involved services/agencies for a particular youth

□ Inclusion of all involved health professionals for a particular youth

□ Identification of a key worker to for each youth to oversee and assist with the process

□ Other – please specify:

**__________________________________________________________________________________________________________________________________________________________________________**

**2. In your experience, what are the most challenging barriers to successful transition to adult services for youth with LTC? Please choose up to 3 that you see as most challenging.**

□ Funding changes from pediatric to adult health services

□ Lack of an identified adult health care provider or service available to provide care

□ Lack of coordinated care

□ Adult providers lack knowledge of pediatric onset conditions

□ Youth/Families feel unprepared

□ Pediatric providers difficulty “letting go” of youth

□ Lack of system integration

□ Lack of continuity of care

□ Types of services and programs change (usually decrease)

□ Remuneration for adult providers does not reflect time required for youth with complex issues

□ Lack of standardized transfer summary

□ Other - please specify:

**__________________________________________________________________________________________________________________________________________________________________________**

**3. Please describe any differences you see in the needs or barriers faced by young people with LTC compared to young people with other types of conditions who transition to adult services.**

**__________________________________________________________________________________________________________________________________________________________________________**

**4. What changes in transition services would you like to see?**

**Small changes:**

**__________________________________________________________________________________________________________________________________________________________________________**

**Larger changes:**

**__________________________________________________________________________________________________________________________________________________________________________**

**5. Please add any further information that you would like to share about the transition process for youth with LTC.**

**__________________________________________________________________________________________________________________________________________________________________________**

**Section D: YOUR BACKGROUND**

**1. What is your professional background?**

□ Physician

□ Nurse

□ Social Worker

□ Occupational Therapist

□ Physical Therapist

□ Other – (Please describe) __________________________________________________

**2. Approximately how many years of experience do you have working with youth with LTC?**

□ <1

□ 1 - 5

□ 6 - 10

□ 11 - 20

□ >20

**3. Indicate the setting where you most commonly work.**

□ Inpatient

□ Outpatient

□ Both inpatient and outpatient

□ Patient’s homes

□ Hospice

**4. Indicate the institution where you most commonly work.**

□ SickKids

□ Bloorview

□ Emily’s House / Philip Aziz

**Thank you for taking the time to complete this survey!**
